# Immunopathogenesis of Severe Acute Respiratory Disease in *Zaire ebolavirus*-Infected Pigs

**DOI:** 10.1371/journal.pone.0061904

**Published:** 2013-04-23

**Authors:** Charles K. Nfon, Anders Leung, Greg Smith, Carissa Embury-Hyatt, Gary Kobinger, Hana M. Weingartl

**Affiliations:** 1 National Center for Foreign Animal Disease, Canadian Food Inspection Agency, Winnipeg, Manitoba, Canada; 2 Special Pathogens Program, National Microbiology Laboratory, Public Health Agency of Canada, Winnipeg, Manitoba, Canada; 3 Department of Medical Microbiology, University of Manitoba, Winnipeg, Manitoba, Canada; Institut National de la Santé et de la Recherche Médicale U 872, France

## Abstract

Ebola viruses (EBOV) are filamentous single-stranded RNA viruses of the family *Filoviridae*. *Zaire ebolavirus* (ZEBOV) causes severe haemorrhagic fever in humans, great apes and non-human primates (NHPs) with high fatality rates. In contrast, *Reston ebolavirus* (REBOV), the only species found outside Africa, is lethal to some NHPs but has never been linked to clinical disease in humans despite documented exposure. REBOV was isolated from pigs in the Philippines and subsequent experiments confirmed the susceptibility of pigs to both REBOV and ZEBOV with predilection for the lungs. However, only ZEBOV caused severe lung pathology in 5–6 weeks old pigs leading to respiratory distress. To further elucidate the mechanisms for lung pathology, microarray analysis of changes in gene expression was performed on lung tissue from ZEBOV-infected pigs. Furthermore, systemic effects were monitored by looking at changes in peripheral blood leukocyte subsets and systemic cytokine responses. Following oro-nasal challenge, ZEBOV replicated mainly in the respiratory tract, causing severe inflammation of the lungs and consequently rapid and difficult breathing. Neutrophils and macrophages infiltrated the lungs but only the latter were positive for ZEBOV antigen. Genes for proinflammatory cytokines, chemokines and acute phase proteins, known to attract immune cells to sites of infection, were upregulated in the lungs, causing the heavy influx of cells into this site. Systemic effects included a decline in the proportion of monocyte/dendritic and B cells and a mild proinflammatory cytokine response. Serum IgM was detected on day 5 and 6 post infection. In conclusion, a dysregulation/over-activation of the pulmonary proinflammatory response may play a crucial role in the pathogenesis of ZEBOV infection in 5–6 weeks old pigs by attracting inflammatory cells to the lungs.

## Introduction

Some of the worst haemorrhagic fever outbreaks in humans, great apes and nonhuman primates (NHPs) have been caused by Ebola viruses (EBOV) and Marburg virus (MARV). These enveloped filamentous, negative sense single-stranded RNA viruses belong to the family *Filoviridae.* The African EBOV species, including *Zaire ebolavirus* (ZEBOV), *Sudan ebolavirus* (SEBOV), *Cote d’Ivoire ebolavirus* (CIEBOV) and *Bundibugyo ebolavirus* (BEBOV) have been associated with disease of varying morbidity and mortality in humans. So far ZEBOV has had the highest case fatality rate ranging from 60 to 90% [1]. The only EBOV species found outside of Africa is *Reston ebolavirus* (REBOV) [2]. Though REBOV can infect humans, it has never been associated with clinical disease in people [1]. However, it can cause disease and mortality in cynomolgus macaques [3].

Until recently, NHPs were considered the only animal species susceptible to REBOV. In 2008, REBOV was detected in pigs co-infected with porcine respiratory and reproductive syndrome virus (PRRSV) in the Philippines. Antibodies to REBOV were also detected in humans that had been in close contact with the infected pigs, suggesting pig to human transmission [4]. Since these pigs were co-infected with PRRSV, it was not clear whether the observed clinical signs were due to REBOV or the co infecting virus. Subsequent experimental challenge suggested that REBOV infection in pigs may be asymptomatic despite replication of the virus in the lungs and shedding in nasopharyngeal secretions [5]. Nevertheless, the emergence of REBOV in pigs had prompted our group to investigate whether the African EBOV species could also infect pigs [6]. Using experimental challenge with the highly pathogenic ZEBOV, we observed clinical disease in pigs in the form of fever, severe respiratory distress characterized by rapid abdominal breathing; inappetence and lethargy [6]. In addition, the infected pigs could transmit virus to contact animals. Gross pathological lesions included pulmonary consolidation, enlarged and sometimes haemorrhagic lung associated lymph nodes. Histopathology lesions consisted of alveolitis characterized by haemorrhage, presence of fibrin, neutrophils and macrophages in alveoli as well as inflammatory cells in thickened alveolar walls. Viral antigen was detected by immunohistochemistry in bronchiolar and alveolar epithelial cells, endothelial cells, macrophages and in inflammatory exudates in trachea. Apparently, experimental ZEBOV and REBOV in pigs seem to target the respiratory system but only the former induces overt clinical disease [5], [6].

In humans and non-human primates (NHPs), ZEBOV causes a severe systemic disease and dysregulated serum proinflammatory cytokine response is often linked to the disease pathogenesis. Changes in peripheral leukocytes subsets have also been observed in human and NHP ZEBOV infections.

The first study indicated local cytokine responses and cellular changes in the lungs of ZEBOV-infected pigs. Proinflammatory cytokines (IFN-γ, IL-6, IL-8 and TNF-α) were induced early while INF-α was significantly down-regulated in these lungs. Cellular changes included the infiltration of neutrophils, macrophages and lymphocytes into alveoli, with macrophages being the only leukocyte subset in the lungs that stained positive for ZEBOV. The aim of the current report was to further elucidate the mechanisms involved in the cellular infiltration into the lungs, by using a porcine microarray to analyze changes in mRNA transcription for a range of proinflammatory cytokines, chemokines and acute phase proteins in porcine lungs. In addition, we aimed to characterize the cellular infiltrates into the lungs using cell type-specific surface markers, also permitting the definitive identification of the cells harbouring ZEBOV antigen. Furthermore, we attempted to evaluated changes in PBMC subsets and serum cytokines.

## Materials and Methods

### Virus

The Kikwit 95 isolate of ZEBOV was produced and titrated on Vero E6 cells as previously described [6].

### Animal Inoculation, Monitoring and Sample Collection

The data presented in this report was generated from 2 experiments. All procedures involving infectious ZEBOV were carried out in containment level 4 (CL4) at the National Center for Foreign Animal Disease, Winnipeg. Animal use was approved by the Canadian Science Centre for Human and Animal Health animal care committee under animal use document (AUD) # C-11-004. The Canadian Council on Animal Care guidelines were followed for all animal manipulations.

#### Experiment 1

The experimental design and part of the data from this experiment has been published [6]. In the current report lung sections from this experiment were used for a porcine microarray to analyse changes in proinflammatory cytokine and chemokine transcription. Briefly, eight 4–5 weeks old pigs were obtained from a commercial farm in Manitoba and allowed an acclimatization period of at least 1 week at NCFAD. These pigs were negative for PRRSV and porcine circovirus -2 (PCV-2). Six pigs were given inhalation anaesthesia and then inoculated with 10^6^ PFU of ZEBOV in sterile PBS per pig via the intranasal, intraocular and oral routes [6]. Two control pigs were mock inoculated with PBS and housed separately. Two ZEBOV-infected pigs were euthanized at 3, 5 and 7 days post infection (DPI) respectively and tissues collected. Mock infected pigs were also euthanized at DPI 7. Lung sections for microarray were stored at −70°C and processed as described subsequently.

#### Experiment 2

Experiment 2 was a repeat of experiment 1 aimed at further investigating the findings in experiment 1 as well as the systemic effects of ZEBOV infection in pigs. Six pigs of the same age group and source as in experiment 1 were acclimatized and inoculated with ZEBOV as described above. Clinical signs were recorded daily. In addition, rectal temperatures were recorded and swabs (oral, rectal and nasal) collected in PBS for each pig before inoculation, and at 1, 3 and 5 or 6 days post inoculation (DPI) under inhalation anaesthesia. Blood was collected from each pig into BD vacutainer CPT™ cell preparation tubes with sodium citrate (Becton Dickinson, New Jersey, USA) at the indicated time points. Three pigs were euthanized at DPI 5 and 3 at DPI 6 and tissues collected for virus isolation and pathology.

### Virus Detection

Virus detection data for experiment 1 has already been published [6]. In experiment 2, the presence of virus in tissues collected at necropsy, blood and swabs was detected by real-time reverse transcriptase polymerase chain reaction (rRT-PCR) targeting the EBOV L-gene. Tissues were homogenized in MEM using a bead mill homogenizer according to the manufacturer’s protocol (Tissue Lyser, Qiagen). Total RNA was extracted from tissues and swabs using Tripure Reagent (Roche; Laval, QC, Canada) according to manufacturer’s protocol. Enteroviral armoured RNA (Asuragen, Austin, Tx., U.S.A.) was used as an exogenous extraction/reaction control. A standard curve generated by serial 10 fold dilutions of EBOV L-gene plasmid was included for every run. The One-Step real-time rRT-PCR was performed using a Quantitect Reverse Transcriptase Real-Time PCR Kit (Qiagen; Toronto, ON., Canada) and the following primers and probe:

ZEBOVForward -CAGCCAGCAATTTCTTCCAT;

ZEBOVReverse- TTTCGGTTGCTGTTTCTGTG;

ZEBOVProbe FAM-ATCATTGGCGTACTGGAGGAGCAG-NFQ.

Reactions were run on a Rotor-Gene 6000 (Qiagen; Toronto, ON., Canada) under the following conditions: 50°C for 30 minutes; 95°C for 15 minutes; 45 cycles of 95°C for 15 seconds followed by 60°C for 45 seconds. Ct values were determined and the EBOV L-gene copy number in each sample calculated based on a standard curve.

### Histopathology and Immunohistochemistry

Hematoxylin and eosin (HE) staining of lung sections from experiment 2 for histopathologic examination and immunohistochemistry (IHC) for ZEBOV VP40 were performed on formalin-fixed paraffin-embedded tissue sections as previously described [6]. Identification of inflammatory cells in the lungs was performed by immunostaining for with the mouse monoclonal antihuman L1 marker antibody Clone Mac387 (Dako, USA) which cross-reacts with porcine macrophages and neutrophils [7]. For epitope retrieval, slides were treated with high pH AR10 (BioGenex, CA) in a BioCare Medical decloaking chamber. Clone Mac 387 (Dako, USA) was applied for 10 minutes at a dilution of 1∶400. It was visualised using a horseradish peroxidase (HRP) labelled polymer, Envision®+system (anti-mouse) (Dako, USA) and reacted with DAB. To identify the presence of T lymphocytes, epitopes were retrieved using glyca solution (BioGenex, CA) in the decloaking chamber and then a polyclonal rabbit anti-human CD3 (Dako, USA) which cross-reacts with porcine CD3 [8] was applied for 60 minutes at a dilution of 1∶200. They were then visualized using a HRP labelled polymer, Envision®+system (anti-rabbit) (Dako, USA) and reacted with 3,3′-Diaminobenzidine (DAB). To identify the presence of B-lymphocytes, epitopes were retrieved using EDTA target retrieval, in a decloaking chamber and a CD79a mouse anti-human monoclonal (clone HM57) (LifeSpan Biosciences, USA) which cross-reacts with porcine CD3 [8] was applied for 60 minutes at a dilution of 1∶2400. They were then visualized using a HRP labelled polymer, Envision®+system (anti-mouse) and reacted with DAB. For the Ebola/Mac387 double stain, tissues were pretreated with proteinase K for 10 minutes. The VP40 antibody was applied to the sections as previously described [6]. The sections were incubated with a denaturing solution (1 part A, 3 parts B, BioCare Medical, CA) for 5 minutes to ensure that the second staining protocol would not react with the first. The slides were then placed in the decloaking chamber in high pH AR10 to retrieve antigens. Clone Mac 387 was applied for 10 minutes at a dilution of 1∶100. An AP-polymer kit, Mach 4 Universal (BioCare Medical, CA) was the applied for 30 minutes and reacted with Vulcan Fast Red (BioCare Medical, CA) substrate. All sections were counterstained with Gill’s hematoxylin.

### Microarray

Lung sections from euthanized control and ZEBOV-infected pigs in experiment 1 [6] were homogenized in MEM using a bead mill homogenizer and total RNA isolated as described above. RNA concentration was determined using a NanoDrop (NanoDrop Technologies). Microarray was performed at the University Health Network Microarray Centre, Toronto, Canada, using an Agilent porcine 4x44K microarray (Agilent Technologies) according to manufacturer’s protocols. Microarray slides were scanned on G2565C DNA scanner and images processed using the Agilent Feature Extraction software version 10.7. Quality of samples was analyzed in R Version 2.10.0 using the ArrayQualityMetrics package version 2.4.3 and data imported into GeneSpring GX version 11.0.1 (Agilent) for further analysis. Data was transformed to log base 2 prior to statistical analysis. First, an expression filter was applied such that probes with at least 1 out of 16 samples having values between 20 and 100 percentile in the raw data were retained for further analysis. One-way ANOVA was performed among the groups and Benjamini and Hochberg FDR method was used for multiple testing correction. Data with corrected p<0.05 and any pair (infected lungs vs control) with fold changes > 2 were considered significant. The complete data set is found in Gene Expression Omnibus (GEO) Database in NCBI under series record GSE44565.

### Peripheral Blood Mononuclear Cells Isolation and Flow Cytometry

Peripheral blood mononuclear cells (PBMCs) were isolated from citrated blood collected from pigs in experiment 2 in BD vacutainer CPT™ cell preparation tubes (Becton Dickinson) according to manufacturer’s protocol. Briefly, the tubes with blood were centrifuged at 1800 g at room temperature for 20 minutes, plasma collected into cryovials and stored at −70°C while the PBMC layer was transferred into a falcon tube. The PBMCs were washed twice in PBS and used for flow cytometry. Approximately 10^6^ cells in 100 µl PBS were also stored at −70°C for subsequent RNA extraction and cytokine RT-PCR.

For flow cytometry, antibodies against porcine surface markers CD3 FITC (SouthernBiotech), CD21 PE (SouthernBiotech), CD14 FITC (AbD Serotec), CD172a PE (SouthernBiotech) and the corresponding isotype controls were used. Approximately 10^6^ PBMCs/tube were resuspended in 100 µl FACS buffer (PBS containing 0.1% bovine serum albumin and 0.1% sodium azide) and antibodies for porcine CD3, CD21, CD14/CD172a (double staining) or isotype controls added to corresponding tubes and incubated on ice for 30 min. After washing twice with FACS buffer, cells were fixed overnight at 4°C in 10% phosphate-buffered formalin before analyzing on the FC500 two laser flow cytometer (Beckman Coulter). At least 25,000 events were acquired per sample and data analysed with the CXP analysis software (Beckman Coulter).

### Quantification of Systemic Cytokine Response

Interferon alpha (IFN-α) levels in plasma of pigs in experiment 2 were measured by ELISA using matched antibody pair from PBL (PBL Interferon Source, Piscataway, NJ, USA) following a previously described protocol [9]. Plasma interleukin 6 (IL-6) was quantified using the porcine IL-6 DuoSet (R&D Systems) according to manufacturer’s protocol.

To measure cytokine mRNA changes in PBMCs collected from pigs in experiment 2, RNA was extracted from approximately 10^6^ PBMCs that were isolated and stored in PBS as described above. The cells were mixed with TriPure Isolation reagent (Roche) and RNA isolated according to the manufacturer’s protocol. Cytokine mRNA transcription was measured by semi quantitative real time RT-PCR using the porcine primers and probes from the TaqMan Gene Expression Assay Library (ABI) with β-actin as the housekeeping gene [6], [10]. Differences in mRNA transcription during infection were then expressed as fold change relative to pre-infection levels [10]. A fold change > 2 was considered significant.

### *In vitro* Infection of PBMC Subsets with ZEBOV

PBMCs were isolated from blood obtained from naïve pigs as described above. The cells were resuspended in RPMI supplemented with 10% fetal bovine serum, 100 U/mL penicillin, 100 µg/mL streptomycin, 200 µM/mL glutamax and 10 mM HEPES (complete medium) and incubated in cell culture flasks at 37°C overnight for monocytes to attach. Non-adherent cells were removed, adherent monocytes washed twice with sterile PBS and then harvested by incubating with TrypLE™ (Invitrogen) at 37°C until the cells detached. The non-adherent cells were sorted by positive selection into CD3+ T cells and CD21+ B cells using cell type specific marker antibodies and antimouse IgG microbeads (MACS, Miltenyi Biotec; Auburn, CA, USA) and the manufacturer’s protocol. Monocytes, B cells and T cells were then exposed to ZEBOV by incubating approximately 5×10^5^ cells with 0.1 MOI of virus in RPMI for 1 h at 37°C. The cells were washed, resuspended in complete medium and incubated at 37°C. After 24 h the supernatant was removed and cells permeabilized using BD cytoperm/cytofix (Becton Dickinson) according to manufacturer’s protocol. Rabbit anti-EBOV VP40 was added at 1/100 dilution in 100 ul of BD cytoperm/cytofix staining/wash buffer, incubated at 4°C for 30 mins, washed twice with cytoperm/cytofix staining/wash buffer and goat anti-rabbit AlexaFluor 594 added at 1/100 and incubated for another 30 mins at 4°C. For control, uninfected cells were stained with anti VP40 in the same manner as the infected cells. Cells were washed twice, formalin fixed and analysed by flow cytometry as described earlier.

### Antibody Detection

An IgM capture ELISA was performed on serum from experiment 2 as previously described [11], [12] using reagents provided by Dr Pierre Rollin from the Center for Disease Control, Atlanta, USA. Briefly, plates were coated with goat anti swine IgM followed by the addition of duplicates of 1∶100 dilution of serum from ZEBOV-infected pigs to allow capture of swine IgM. ZEBOV antigen was then added to bind to captured antigen-specific IgM. Subsequently, rabbit polyclonal anti-EBOV IgG was added to bind ZEBOV antigen and this reaction was detected by adding peroxidase-conjugated antibody against rabbit IgG followed by ABTS substrate. Optical density (OD) was determined at 405 nm. The average OD_405_ for negative control antigen was subtracted from that of the corresponding ZEBOV antigen to obtain the adjusted OD_405_ for each serum sample. The cut-off value above which samples were considered positive for IgM was determined as the mean +3 standard deviation of OD_405_ for negative control sera.

### Statistical Analysis

Data from multiple timepoints was analyzed by ANOVA and Dunnett’s multiple comparisons post test, using GraphPad Prism version 5. Microarray data was analyzed by one-way ANOVA, and Benjamini and Hochberg FDR method was used for multiple testing correction. p<0.05 was considered statistically significant.

## Results

### Clinical Signs and Viral RNA Detection

The clinical signs in ZEBOV-infected pigs in experiment 1 and 2 were as previously described [6]. These included fever starting 4 days post-inoculation (DPI). At the same time, there was an increase in respiratory rate as well as difficult, abdominal breathing, inappetence, weakness and reluctance to move.

Viral RNA detection reported here is for pigs in experiment 2. The results for pigs in experiment are already published [6]. Viral RNA was detected by rRT-PCR in oral swabs from all pigs beginning on DPI 1 (2 pigs), DPI 3 and 5 (4 pigs) (Table 1). A subset of pigs also had viral RNA in nasal swabs on DPI 1, 5 and 6. All rectal swabs were negative for ZEBOV RNA. Only 2 pigs had detectable viremia based on RNA detection on DPI 5 (1 pig) and DPI 6 (1 pig) (Table 1).

**Table 1:** Virus detection in blood and swabs from pigs infected with *Zaire ebolavirus* (ZEBOV).

	Blood				Oral swab				Nasal swab			
	DPI 1	DPI 3	DPI 5	DPI 6	DPI 1	DPI 3	DPI 5	DPI 6	DPI 1	DPI 3	DPI 5	DPI 6
Pig 7	U	U	U	U	U	U	3.7 (0.01)	3.2 (0.014)	U	U	3.98 (0.127)	6.1 (0.042)
Pig 8	U	U	U	3.91 (0.0002)	3.6 (0.21)	U	U	U	4.8 (0.007)	U	3.7 (0.12)	3.94 (0.0)
Pig 9	U	U	U	U	U	4.18 (0.076)	U	U	U	U	U	U
Pig 10	U	U	U		4 (0.163)	4.1 (0.021)	4.3 (0.012)		U	U	U	
Pig 11	U	U	U		U	2.4 (0.057)	4.15 (0.029)		U	U	U	
Pig 12	U	U	3.86 (0.0005		U	4.5 (0.0)	3.4 (0.021)		U	U	U	

Virus detection for pigs infected with *Zaire ebolavirus* in experiment 2 was done by quantitative real time RT-PCR targeting the L gene of ZEBOV and L gene copy numbers estimated based on a standard curve. Data represents log_10_ copies/mL of blood or swab solution (numbers in brackets represent standard deviations of 3 replicates for each sample). DPI, days post infection; ND, not done; U, virus was either absent or below levels detectable by the current assay.

In addition, viral RNA was detected in submandibular lymph nodes of all pigs, and in bronchial lymph nodes, lungs and trachea of all but one pig, with the lungs having the highest mean levels of viral RNA (Table 2). Half of the animals had detectable virus RNA in the spleen. However, no viral RNA was detected in muscle and only one pig had virus RNA in the mesenteric lymph nodes (Table 2).

**Table 2:** Virus detection in tissues from pigs infected with *Zaire ebolavirus* (ZEBOV).

	Day Euthanized	SLN	BLN	MLN	Lung	Liver	Spleen	Trachea	Muscle
Pig 7	6	4.95 (0.014)	5.99 (0.065)	U	3.73 (0.244)	4.17 (0.064)	U	4.39 (0.111)	U
Pig 8	6	5.44 (0.58)	9.52 (0.012)	4.47 (0.075)	9.56 (0.678)	5.94 (0.006)	5.92 (0.0)	4.42 (0.035)	U
Pig 9	6	U	7 (0.002)	U	9.54 (0.583)	4.94 (0.01)	4.39 (0.1)	5.94 (0.012)	U
Pig 10	5	5.43 (0.04)	7.92 (0.002)	U	9.37 (0.46)	3.62 (0.105)	U	4.94 (0.038)	U
Pig 11	5	5.5 (0.29)	U	U	U	U	U	U	U
Pig 12	5	5.94 (0.01)	7.01 (0.134)	U	7.53 (0.52)	5.36 (0.141)	5.53 (0.017)	5.57 (0.032)	U

Virus detection for pigs infected with *Zaire ebolavirus* in experiment 2 was done by quantitative real time RT-PCR targeting the L gene of ZEBOV and L gene copy numbers estimated based on a standard curve. Data represents log_10_ copies/mL of tissue suspension (numbers in brackets represent standard deviations of 3 replicates for each sample). SLN, submandibular lymph node; BLN, bronchial lymph node; MLN, mesenteric lymph nodes, U, virus was either absent or below levels detectable by the current assay.

### Gross Pathology and Histopathology

Gross pathology was most prominent in the lungs. The pneumonia was distributed primarily in the dorso-caudal lobes and was characterized by consolidation and haemorrhage affecting more than 70% of the lung tissue.

**Figure 1:**
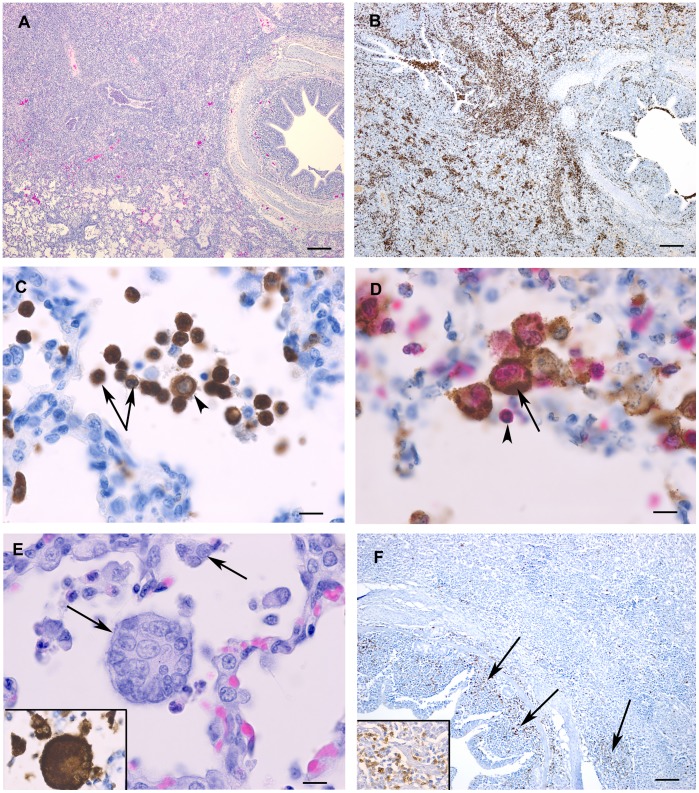
Histopathology and immunohistochemistry findings in *Zaire ebolavirus* (ZEBOV) -infected pig lungs. (A). Hematoxylin and eosin stained slide showing infiltration of inflammatory cells into affected lobules and some fluid-filled alveoli. Bar = 200 µm. (B) The majority of the inflammatory cells within the lungs showed positive immunostaining with antibody Mac387 against L1 antigen. Bar = 200 µm. (C) Higher magnification of (B) showing several neutrophils (arrows) and a macrophage (arrowheads). Bar = 10 µm. (D) Double immunolabelling detected the simultaneous expression of Mac387 macrophage marker (pink) and ZEBOV antigen (brown, arrows) within the same cell indicating the presence of viral antigen within macrophages. Viral antigen was not detected within neutrophils (arrowhead). Bar = 10 µm. (E) Numerous multinucleated syncytial cells (arrow) were observed within alveolar spaces. H&E stain. Bar = 10 µm. *Inset:* Serial section of lung with specific immunolabelling for ZEBOV antigen in multinucleated syncytial cells (arrows) and mononuclear inflammatory cells (arrowhead). (F) CD3 positive T-lymphocytes were limited to infiltrates in the bronchiolar lamina propria and peribronchial areas (arrows). *Inset:* Higher magnification of perbronchiolar CD3 positive T-lymphocytes. Lung sections were obtained from pigs in experiment 2.

There was inflammation and necrosis in the bronchial lymph nodes and mild tracheitis. The lung lesions were characterized by bronchointerstitial pneumonia with accumulation of neutrophils, macrophages and necrotic debris in alveolar and bronchiolar lumens and peribronchiolar/perivascular infiltration of inflammatory cells (Figure 1A). Approximately 80% of the inflammatory cells in the lungs showed positive immunostaining using Clone Mac387 macrophage and neutrophil marker (Figure 1B) and could be identified as primarily neutrophils with slightly lesser numbers of macrophages based on morphology (Figure 1C). Viral antigen was detected extensively in alveolar and septal macrophages using double immunostaining (Fig. 1D). A previously unreported feature was the presence of numerous multinucleated cells within alveoli which show positive immunostaining for Ebola virus antigen using IHC (Fig. 1E). Viral antigen was widely distributed, often observed in a peribronchiolar pattern and could be detected within inflammatory cells, type II pneumocytes, endothelial cells and bronchiolar epithelial cells. Immunostaining for CD3 showed that the presence of T lymphocytes was limited to bronchiolar submucosa and peribronchiolar areas and represented only a small proportion of the inflammatory infiltrate in the lungs (Figure 1F). Ebola virus antigen could not be detected by IHC in areas of T lymphocyte infiltration. Immunostaining for CD79a showed only few scattered B-lymphocytes throughout the section (not shown).

### Microarray Data

The complete microarray data is found in Gene Expression Omnibus (GEO) Database in NCBI under series record GSE44565. Changes in gene expression in lung sections from infected pigs in experiment 1 relative to control pigs were minimal at DPI 3. Higher fold changes were observed at DPI 5 and 7. Emphasis was placed on proinflammatory cytokines, chemokines and associated genes to explain the inflammatory cell infiltrate into the lungs.

Proinflammatory cytokines involved in activation and recruitment of immune cells to the site of infection (IL-5, IL-6, IL-8, IL-22, IL-26 and IL-27, SELL, RETN and PLUNC) and the anti-inflammatory cytokine IL-10 genes were up-regulated in ZEBOV-infected lungs (Table 3). Chemokine genes implicated in attraction of immune cells to site of infection including CCL2, CCL10, CCL19, CCL20, AMCF-II (granulocytes), CCL3L1 and CCL4 (neutrophils), were up-regulated starting on DPI 3 (Table 4). The mRNA for the acute phase protein, SAA, was also up-regulated in the lungs of ZEBOV-infected pigs. In addition, the transcriptome for molecules involved in the apoptosis pathway (the caspases, CARD6, AATYK, FAS, FADD, TRAF3 and TNIP3) was also up-regulated in ZEBOV-infected lung (Table 5). Transcription of genes for pattern recognition receptor (PRR), transcription factors and interferon-stimulated genes (ISG) was up-regulated in ZEBOV-infected lungs from DPI 3- 7 (Table 6).

**Table 3:** Cytokine response in lungs from pigs infected with *Zaire ebolavirus.*

Gene	Description	Fold change			Function
		DPI 3	DPI 5	DPI 7	
IL-6	Interleukin- 6	2	41	23	Recruitment of inflammatory cells; induction of acute phase proteins
IL-8	Interleukin-8	8	106	45	
IL-22	Interleukin-22	1	59	30	
IL-5	Interleukin- 5	3	4	5	Attracts eosinophils
IL-26	Interleukin-26	1	10	5	Mucosal & cutaneous immunity
IL-27	Interleukin-27	2	37	48	Drives expansion of CD4 T cells and IFN-γ secretion
GM-CSF	Granulocyte monocyte colony stimulating factor	2	25	16	Controls production, differentiation & function of granulocytes & monocytes
SPP1	Secreted phosphoprotein 1	3	37	36	Regulation of IL-12 & IL-10 secretion
SELL	Selectin	4	57	97	Cell adhesion and trafficking
RETN	Resistin	1	248	185	Proinflammatory by inducing TNF-α & IL-12 from macrophages
PLUNC	palate, lung and nasal epithelium associated	66	496	273	Airway inflammation, innate immunity
IL-10	Interleukin-10	2	20	18	Anti inflammatory

Data was obtained by microarray analysis of RNA from lungs of pigs infected with *Zaire ebolavirus* in experiment 1. Numbers represent fold change in transcription relative to tissues from uninfected controls. DPI, days post infection. N = 2 pigs for controls, DPI 3, 5 and 7.

**Table 4:** Chemokine and acute phase response in lungs from pigs infected with *Zaire ebolavirus.*

Gene	Description	Fold change			Function
		DPI 3	DPI 5	DPI 7	
CCL2	Chemokine (C-C motif) ligand 2	1	29	32	Chemoattractant for immune cells
CCL3L1	Chemokine (C-C motif) ligand 3-like 1	2	21	22	
CCL4	Chemokine (C-C motif) ligand 4	2	33	28	
CCL10	Chemokine (C-C motif) ligand 10	10	109	211	
CCL19	Chemokine (C-C motif) ligand 19	2	12	9	
CCL20	Chemokine (C-C motif) ligand 20	7	12	13	
AMCF-II	Alveolar macrophage-derived chemotactic factor	2	478	258	
SAA	Serum amyloid A2	4	907	836	
C9	Complement component 9	4	48	72	Cytolysis via pore formation

Data was obtained by microarray analysis of RNA from lungs of pigs infected with *Zaire ebolavirus* in experiment 1. Numbers represent fold change in transcription relative to tissues from uninfected controls. DPI, days post infection. N = 2 pigs for controls, DPI 3, 5 and 7.

**Table 5:** Induction of proapoptotic molecules in lungs from pigs infected with *Zaire ebolavirus.*

Gene	Description	Fold change		
		DPI 3	DPI 5	DPI 7
CASP1	Caspase 1	2	13	12
CASP3	Caspase 3	1	3	3
CASP1/4	Caspase 1/3	2	6	5
CASP8	Caspase 8	1	3	2
CASP15	Caspase 15	1	3	5
CARD6	Caspase recruitment domain family	2	4	4
AATYK	Apoptosis-associated tyrosine kinase 2	1	4	4
FADD	Fas (TNFRSF6)-associated via death domain	2	3	3
FAS	TNF receptor superfamily, member 6	2	5	5
TRAF3	TNF receptor-associated factor 3	2	6	7
TNIP3	TNFAIP3 interacting protein 3 isoform 3	3	10	14
AIF1	Allograft inflammatory factor 1	1	3	3

Data was obtained by microarray analysis of RNA from lungs of pigs infected with *Zaire ebolavirus* in experiment 1. Numbers represent fold change in transcription relative to tissues from uninfected controls. DPI, days post infection. N = 2 pigs for controls, DPI 3, 5 and 7.

**Table 6:** Up-regulation of pattern recognition receptors, transcription factors and interferon stimulated genes in lungs from pigs infected with *Zaire ebolavirus.*

Gene	Description	Fold change			Function
		DPI 3	DPI 5	DPI 7	
CD14	CD14 molecule	1	40	62	Coreceptor for TLR4 signalling
CD163	CD163 molecule	8	64	95	Pattern recognition receptors (innate immunity)
TLR2	Toll-like receptor 2	3	36	41	
TLR4	Toll-like receptor 4	4	22	21	
TLR6	Toll-like receptor 6	2	6	5	
TREM-1	Triggering receptor expressed on myeloid cell -1	5	81	77	Proinflammatory
STAT1	Signal transducer & activator of transcription 1	3	20	26	Signal transduction, antiviral response
STAT2	Signal transducer & activator of transcription 2	2	4	4	
STAT5A	Signal transducer & activator of transcription 5A	3	4	8	
IRF1	interferon regulatory factor 1	2	6	6	Innate immunity/antiviral response
IRF7	interferon regulatory factor 7	4	15	20	
DDX58 (RIG-I)	Retinoic acid inducible protein I	12	77	253	
ISG15	Interferon-induced 15 kDa protein	20	36	40	
ISG20	Interferon-stimulated gene 20 kDa protein	15	126	143	
IFIH1	Interferon induced with helicase C domain 1	3	12	18	
vLIG-1	Interferon-inducible very large GTPase 1	2	14	17	
IFIT-1	Interferon induced with tetratricopeptide 1	8	11	21	
MX1	Myxovirus (influenza virus) resistance 1	15	36	71	
MX2	Myxovirus (influenza virus) resistance 2	8	12	12	
OASL	2′5′-oligoadenylate synthetase-like	9	32	64	
OAS2	2′5′-oligoadenylate synthetase 2	5	16	47	
IRG6	Inflammatory response protein 6	33	25	37	

Data was obtained by microarray analysis of RNA from lungs of pigs infected with *Zaire ebolavirus* in experiment 1. Numbers represent fold change in transcription relative to tissues from uninfected controls. DPI, days post infection. N = 2 pigs for controls, DPI 3, 5 and 7.

**Figure 2:**
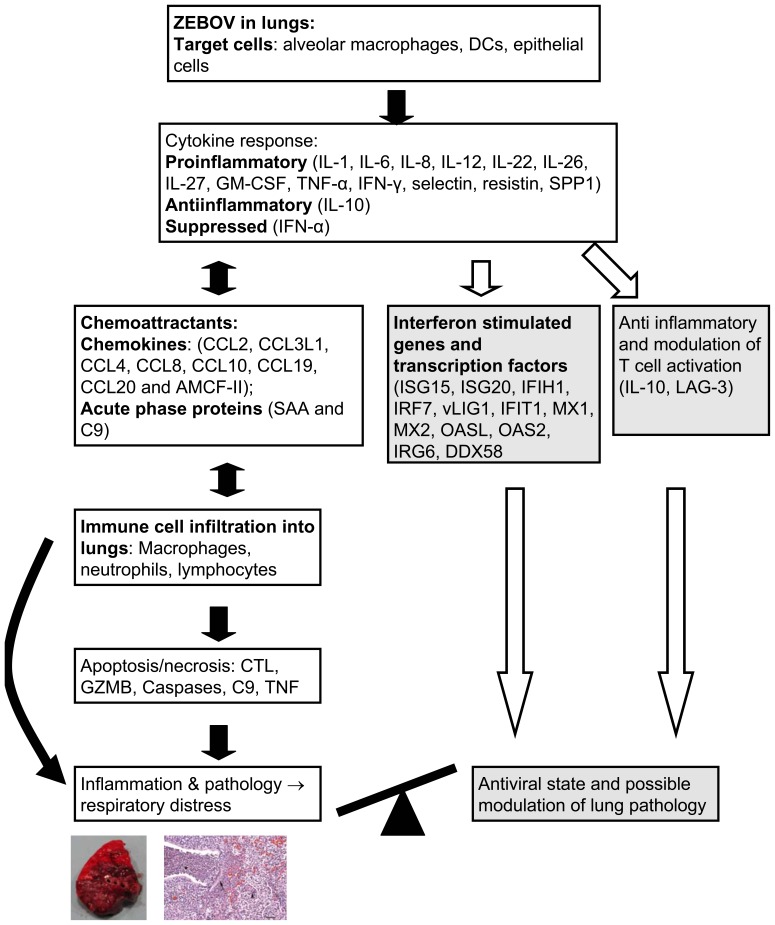
Model for pulmonary disease pathogenesis in *Zaire ebolavirus* (ZEBOV)-infected pigs based on microarray data. Arrows show the proposed sequence of events that take place from the time of ZEBOV infection to the development of lung inflammation and respiratory distress. The grey shaded blocks represent the anti-viral/anti-inflammatory machinery. The scale suggests that the series of events favour the proinflammatory pathway.

Figure 2 shows a proposed model for the pathogenesis of pulmonary distress in ZEBOV-infected pigs.

### Changes in PBMC Subsets

**Figure 3:**
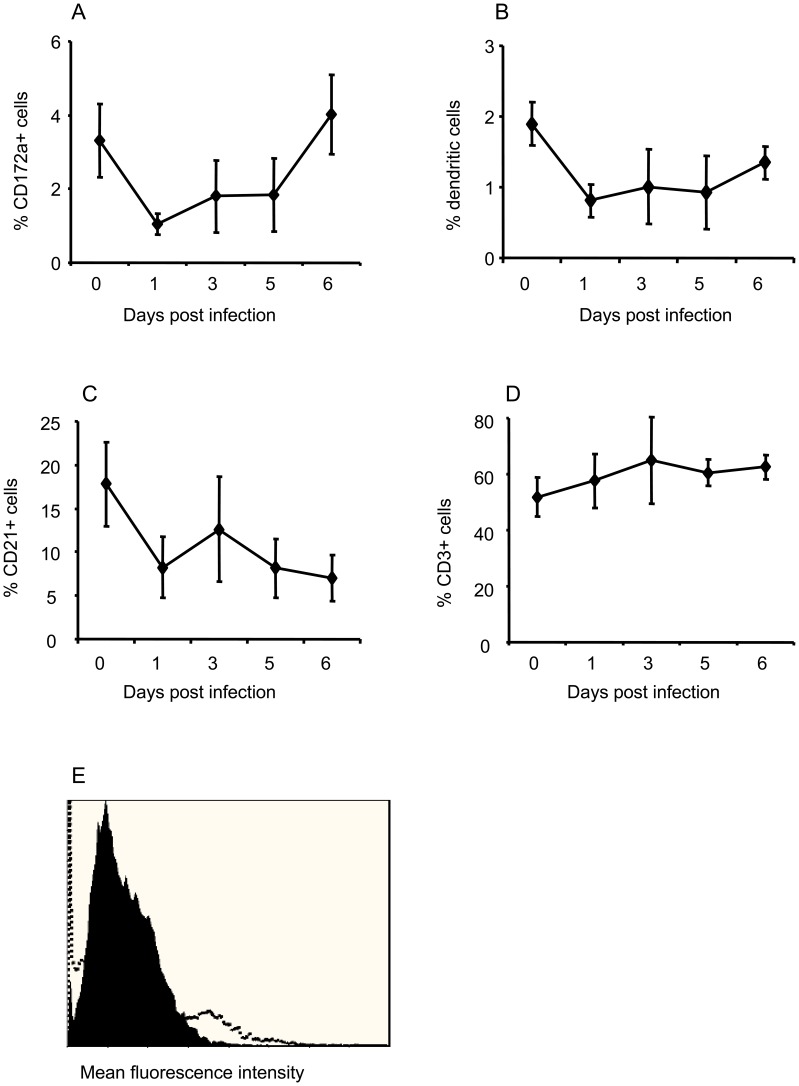
Changes in PBMC subsets in *Zaire ebolavirus* (ZEBOV)-infected pigs. (A) CD172a+ (monocytes/macrophages and dendritic) cells (B) CD14− CD172a+ (dendritic) cells, (C) CD21+ B lymphocytes and (D) CD3+ T lymphocytes in PBMCs. Data points for (A–D) represent means ± standard deviation for n = 6 pigs on 0, 1, 3 and 5 days post infection (DPI) and n = 3 pigs on DPI 6 from experiment 2. (E) a representative plot showing infection of porcine monocytes with ZEBOV determined by flow cytometry. Filled histogram represents the uninfected cells and the open histogram for ZEBOV-infected monocytes for n = 3 different PBMC donor pigs.

The effect of ZEBOV infection of pigs in experiment 2 on PBMC subsets was analyzed by flow cytometry. There was a decline in the proportion of CD172a+ (monocytes/dendritic) cells in PBMCs, reaching statistical significance on DPI 1 and 3 (p<0.05), followed by a rebound on DPI 6 (Figure 3A). Porcine dendritic cells identified as CD172a+ CD14- [13] also significantly declined in PBMCs (p<0.05 for DPI 1, 3 and 5) and remained low till the end of the experiment (Figure 3B). In addition, CD21+ B cells decreased in frequency from DPI 1–6 (Figure 3C), with statistical significance on DPI 1, 5 and 6 (p<0.05). On the contrary, there was no effect on CD3+ T cell frequencies in PBMCs (Figure 3D).

To investigate if the observed decline in cell frequencies was due to direct infection of these cells by ZEBOV, sorted monocytes, B cells and T cells from naïve pigs were exposed to the virus in vitro. Approximately 7–11% of monocytes from 3 different pigs were positive for ZEBOV by intracellular staining and flow cytometry (Figure 3E). Neither B nor T cells were infected (data not shown).

### Systemic Cytokine Response

**Figure 4:**
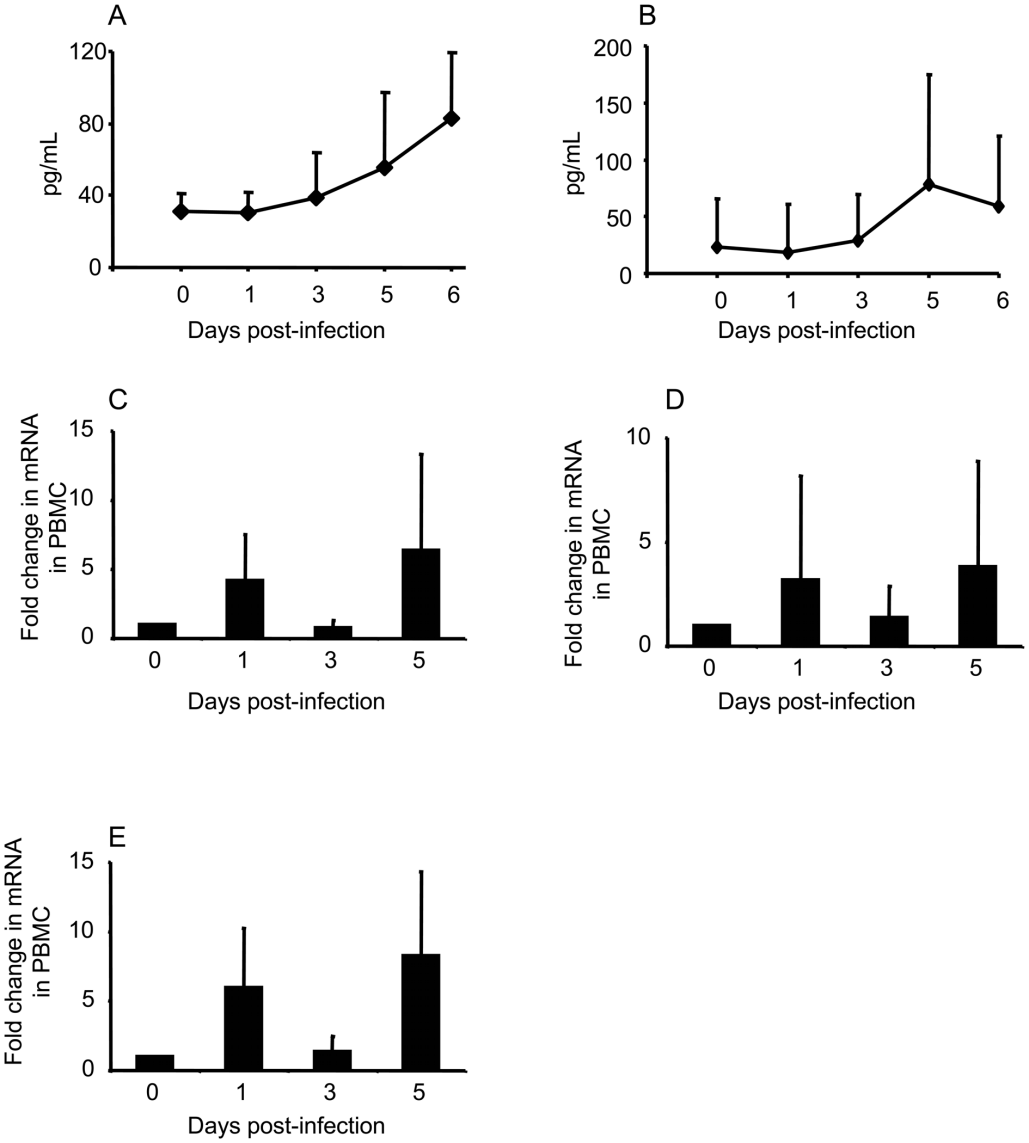
Systemic cytokine response in *Zaire ebolavirus* (ZEBOV)-infected pigs. (A) Serum IFN-α, and (B), serum IL-6 response were measured by ELISA. Data points represent means ± standard deviation for n = 6 pigs on 0, 1, 3 and 5 days post infection (DPI) and n = 3 pigs on DPI 6 from experiment 2. Histograms represent means ± standard deviation of fold change in mRNA transcripts for (C) IL-12; (D) TNF-α, and (E) IFN-γ in PBMCs (n = 6 pigs) from experiment 2.

Systemic cytokine response in ZEBOV-infected pigs in experiment 2 was measured by both ELISA and qRT-PCR. Pigs responded to ZEBOV infection by secreting IFN-α and IL-6 in blood starting at DPI 3 (Figure 4 A & B). Peak levels of IFN-α were attained on DPI 6 (p<0.05 relative to baseline levels). However, the IL-6 response did not reach statistical significance. There was also induction of IL-12, TNF-α and IFN-γ mRNA transcription on DPI 1 and 5 (Figure 4 C–E). The increase in IFN-γ mRNA transcription was statistically significant at DPI 5 (p<0.05).

### Antibody Response

**Figure 5:**
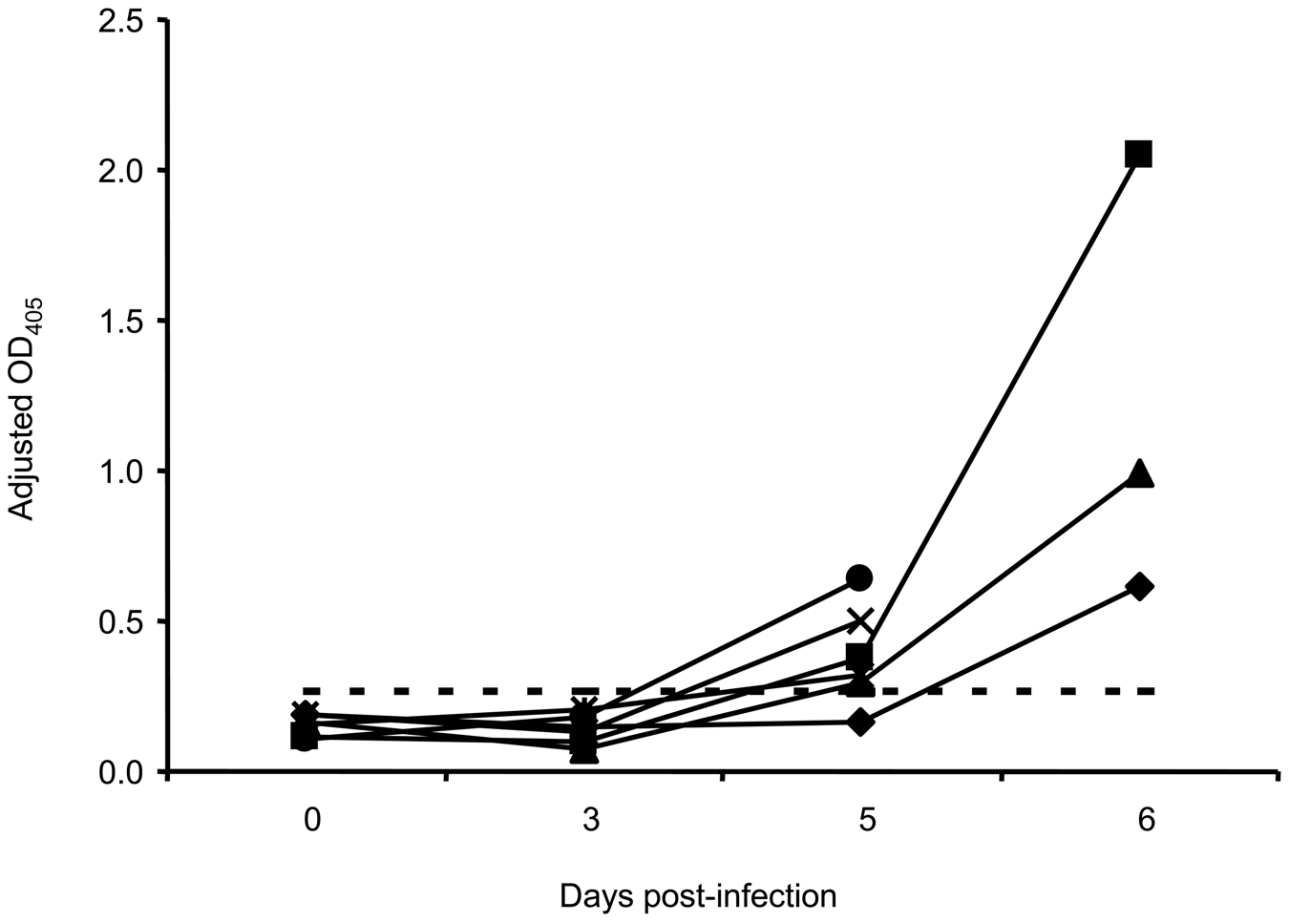
IgM antibody response in *Zaire ebolavirus* (ZEBOV)-infected pigs. IgM was detected by a porcine IgM capture ELISA using serum from pigs in experiment 2. Data for individual animals is presented as adjusted OD_405_ obtained by subtracting the OD_405_ of a negative antigen from that of the ZEBOV antigen for 1/100 dilution of each serum sample. The horizontal broken line represents the cut-off OD_405_ value defined as mean +3 standard deviation of negative control sera.

Serum antibody response was analysed for pigs in experiment 2. Significant increase in IgM antibody levels based on OD_405_ was observed on DPI 5 and 6 (p<0.05, Figure 5). All but 1 pig had OD_405_ values above the cut-off at DPI 5.

## Discussion

To further understand the pathogenesis of disease due to ZEBOV infection in pigs, we have analysed an array of proinflammatory cytokines, chemokines, acute phase proteins and other molecules that could mediate inflammation in the lungs. In addition, we also analysed possible systemic changes due to ZEBOV infection in pigs. The clinical course and pathology in 5–6 week old pigs confirmed that ZEBOV causes a mainly respiratory syndrome in these animals, which can be very severe in some cases [6]. Apparently, REBOV and ZEBOV have the same predilection site in pigs, but the former doesn’t cause clinical disease [5], [6].

Using specific surface markers, we have confirmed that alveolar macrophages were the only infiltrating leukocyte subset in the lungs susceptible to ZEBOV. The susceptibility of human/NHP macrophages to EBOV is well documented [14]. However, to the best of our knowledge, the presence of multinucleated cells within alveoli in the current report is a first in EBOV infection of any species. These cells could have possibly been in the lungs prior to ZEBOV infection since these pigs were not specific pathogen free. Nevertheless, the fact that the cells were positive for ZEBOV antigen would suggest that these giant cells could be a consequence of ZEBOV infection. Similar giant cells believed to be of macrophage lineage, were present in human lungs in fatal cases of severe acute respiratory syndrome but absent in ante-mortem samples [15], [16]. Interestingly, fatal ZEBOV infection in pigs manifests as a severe acute respiratory disease.

We have revealed the induction of proinflammatory cytokines in lungs of ZEBOV-infected pigs. A network of cytokines, chemokines and acute phase proteins possibly interacted in the lungs of these pigs culminating in severe inflammation as suggested in our model in Figure 2. This inflammatory process possibly occurred in a cyclical fashion, with an initial induction of cytokines in resident alveolar cells, followed by the attraction of more cells into the lungs and then further amplification of the inflammatory process. For example, human neutrophils exposed to EBOV *in vitro*, though not infected, rapidly activate triggering receptor expressed on myeloid cells-1 (TREM-1) [17] which results in the release of proinflammatory cytokines and chemokines that in turn attract more cells to the site via induction of vasodilation and increased vascular permeability [14]. Specifically, IL-8 is a strong attractant for neutrophils [18] while IL-5 is known to recruit and activate eosinopils at sites of inflammation [19]. Meanwhile, IL-22 activates IL-6 secretion and both cytokines are potent inducers of acute phase proteins which are also chemoattractants for inflammatory cells [20], [21]. In addition, alveolar macrophage-derived chemotactic factor (AMCF-II) acts as a chemoactractant for neutrophils, eosinophils and basophils to the lungs [18], [22]. Other chemokines and chemokine ligands, most of which are induced by proinflammatory cytokines, also act as chemoattractants for these cells [23]. Furthermore, selectins enable cell adhesion and extravasation, thereby facilitating cell migration to sites of inflammation [24]. Moreover, the high and rapid upregulation of palate, lung and nasal epithelium associated (PLUNC) gene, a marker of airway inflammation [25], conforms to the degree of inflammation observed in the lungs of ZEBOV-infected pigs. In addition, the increase in transcripts of markers (CD14 and CD163) and PRRs expressed on macrophages appears to correlate with the increased presence of these cells within the lungs. Similar proinflammatory cytokine and chemokine responses leading to infiltration of neutrophils, macrophages and other leukocytes into the lungs were linked to respiratory distress in pigs infected with highly pathogenic PRRSV [26], hence the clinical picture observed in pigs co-infected with PRRSV and REBOV in the Philippines in 2008 [4].

Disintegrating cells were observed in bronchioles and alveolar septae of ZEBOV-infected pigs in experiment 1 [6]. Considering the up-regulation of caspases and related molecules, the apoptosis pathway is apparently activated in these lungs. Although apoptosis is a programmed cell death designed to eliminate viral infection [26], the massive cell death would also compromise lung structure and function. Furthermore, caspases mediate inflammation [27].

On the other hand, IL-10 which is a negative regulator of inflammation [21], was slightly up-regulated in the lungs of ZEBOV-infected pigs. This would suggest an attempt to regulate inflammation in the lungs. Furthermore, genes involved in activation of transcription factors and ISGs and subsequently, innate immunity were up-regulated indicating a potential antiviral state. Therefore, pulmonary disease in ZEBOV infection in pigs is most likely a consequence of a dysregulation and/or overstimulation of proinflammatory immune response as shown in our model where the scale is tipped in favour of inflammation (Figure 2).

How does the EBOV-induced lung disease in pigs compare to primates, including humans? Apparently, the same cytokine profile, including IL-6, IL-8, IFN-γ, IL-10 and chemoattractants, exists in both porcine and human/NHP ZEBOV infection. In both cases disease is linked to dysregulation of the proinflammatory response [1], [6], [14], [17], [28]. However, a major difference between ZEBOV in pigs and humans/NHPs is the site of the cytokine response. In pigs the response is more localized in the lungs with very mild systemic involvement while in humans/NHPs it is predominantly systemic. In addition, the high induction of tissue specific chemoactractants such as PLUNC [25] and AMCF-II [22] seems to further favour a severe pulmonary involvement in pigs. Nevertheless, difficult and rapid breathing have been observed in ZEBOV-infected human patients but were associated to underlying metabolic defects rather than pulmonary pathology [29], [30]. Furthermore, a chimpanzee naturally infected with CIEBOV had no gross pathological lesions and only few antigen positive alveolar macrophages in the lungs [31]. However, when rhesus monkeys were aerosol exposed to ZEBOV, they developed what was described as a bronchocentric pattern of interstitial pneumonia. Additionally, there was an increase in number of leukocytes including antigen-positive alveolar macrophages [32]. However, as opposed to what we have observed in pigs, the leukocytosis in monkeys was diffuse [32]. Indeed, we also noticed that although alveolar macrophages were the only leukocyte subset positive for ZEBOV in both NHPs and pigs, there was a significant difference between the 2 species in the type and numbers of cells infiltrating the lungs [33]. Macrophages were the main cells in NHP lungs as opposed to pigs that had a high density of both macrophages and neutrophils in their lungs. The underlying mechanisms for these differences are the subject of further investigation. Differences in type and range of proinflammatory cytokines and chemokines induced in the lungs might be a factor. Note that NHPs challenged with EBOV by both parenteral or aerosol routes develop systemic disease involving numerous organs [32]. This is clearly different in pigs in which induction of proinflammatory molecules and disease is mainly in the lungs, with less pronounced systemic involvement.

Nevertheless, a mild systemic effect of ZEBOV infection in pigs is supported by the depletion of blood monocytes, DC and B cells. The mechanism for this depletion is not entirely clear. As in human/NHP, the direct infection of monocytes by EBOV could lead to cell death [34], [35]. Similar to the reports in humans/NHPs, porcine B and T lymphocytes are not infected by ZEBOV. The apparent decline in B cells and no effect on T cells in peripheral blood of infected swine is contrary to observations in humans/NHPs. Depletion of T cells and not B cells as a consequence of bystander apoptosis is seen in human/NHPs [1]. The B cell depletion in infected pigs might also be due to bystander apoptosis; however additional mechanisms such as migration into spleen and lymph nodes could be involved.

In conclusion, this report sheds more light on the pathogenesis of disease caused by ZEBOV infection in 5–6 weeks old pigs and shows that a series of proinflammatory events occurs, culminating in the infiltration of immune cells into the lungs, leading to severe respiratory distress clinically similar to highly pathogenic PRRSV.
